# Short- and Long-Term Prediction of the Post-Pubertal Mandibular Length and *Y*-Axis in Females Utilizing Machine Learning

**DOI:** 10.3390/diagnostics13172729

**Published:** 2023-08-22

**Authors:** Matthew Parrish, Ella O’Connell, George Eckert, Jay Hughes, Sarkhan Badirli, Hakan Turkkahraman

**Affiliations:** 1 Department of Orthodontics and Oral Facial Genetics, Indiana University School of Dentistry, Indiana University Purdue University at Indianapolis, Indianapolis, IN 46202, USA; mattparr@iu.edu (M.P.); jayhughe@iu.edu (J.H.); 2 Indiana University School of Dentistry, Indiana University Purdue University at Indianapolis, Indianapolis, IN 46202, USA; ellahick@iu.edu; 3 Department of Biostatistics and Health Data Science, Indiana University School of Medicine, Indianapolis, IN 46202, USA; geckert@iu.edu; 4 Eli Lilly and Company, Indianapolis, IN 46225, USA; s.badirli@gmail.com

**Keywords:** artificial intelligence, neural network, regression algorithm, growth and development, mandible

## Abstract

The aim of this study was to create a novel machine learning (ML) algorithm for predicting the post-pubertal mandibular length and *Y*-axis in females. Cephalometric data from 176 females with Angle Class I occlusion were used to train and test seven ML algorithms. For all ML methods tested, the mean absolute errors (MAEs) for the 2-year prediction ranged from 2.78 to 5.40 mm and 0.88 to 1.48 degrees, respectively. For the 4-year prediction, MAEs of mandibular length and *Y*-axis ranged from 3.21 to 4.00 mm and 1.19 to 5.12 degrees, respectively. The most predictive factors for post-pubertal mandibular length were mandibular length at previous timepoints, age, sagittal positions of the maxillary and mandibular skeletal bases, mandibular plane angle, and anterior and posterior face heights. The most predictive factors for post-pubertal *Y*-axis were *Y*-axis at previous timepoints, mandibular plane angle, and sagittal positions of the maxillary and mandibular skeletal bases. ML methods were identified as capable of predicting mandibular length within 3 mm and *Y*-axis within 1 degree. Compared to each other, all of the ML algorithms were similarly accurate, with the exception of multilayer perceptron regressor.

## 1. Introduction

Mandibular growth is a particularly important issue for orthodontists when treating a growing patient. Within the craniofacial complex, there is no component with greater postnatal growth potential than the mandible [1]. Much attention has been given to the growth of the mandible and its unique features stemming from its functional roles [2,3,4], dual growth centers [5], muscular attachments [6], adaptability [7], and interactions with dental structures [8,9]. The magnitude of growth in the mandible also varies with the age of the individual, with the largest changes occurring during and beyond the adolescent growth spurt [10]. There are also very clear gender differences in mandibular growth. Growth differences in the mandible between males and females can be observed in early childhood and become more prominent during adolescence. Girls have been observed to begin, reach peak, and complete the pubertal growth spurt approximately two years prior to their male counterparts [11]. There is also significant difference in the magnitude of growth between the sexes. While females tend to reach the pubertal growth spurt earlier than males, males are shown to have a more intense growth spurt as well as two additional years of growth [12]. The rate of condylar change during peak growth is significantly greater for males, and the overall magnitude change in mandibular length is greater in males than females [13].

Researchers and orthodontists have been attempting to develop methods to predict mandibular growth for decades. Despite these efforts, even the most experienced clinicians often fail on their predictions [14]. Bjork developed a method using metallic implants and cephalometric radiographs to analyze the growth pattern [10] and rotation [15] of the mandible. He suggested that the most accurate way to predict the rotation of the mandible from a single radiograph could be based on seven structural signs that represent bony remodeling of the mandible during growth [15,16]. The predictive value utilizing this method, however, was shown to be no more accurate than inputting random values and therefore deemed clinically unacceptable [17]. Alternatively, Ricketts postulated that the mandible grows along an arc, and growth can therefore be forecasted based on an arcial pattern [18]. 

Mathematical and statistical procedures that build upon the previously described models have also been attempted for predicting mandibular growth. Buschang et al. [19] attempted to use multilevel models that took into consideration the mean growth curve of the population as well as variations in the individuals observed. The multilevel models were compared with individual data extrapolated from growth curves, and no statistically significant benefit was found. In another study attempting to develop a mathematical model, Oueis et al. [20] postulated that evaluating a younger population might lead to a more predictable method. The study evaluated 15 measurements from the lateral cephalograms of children aged 4–9 years old and derived a multiple regression equation that was shown to be of little predictive value.

A fast-growing technology that is being utilized in many fields is that of artificial intelligence (AI) and machine learning (ML). For simple AI to predict an outcome, it requires every possible outcome to be programmed into its algorithm. ML is a subset of AI that eliminates this requirement and allows the computer to learn from inputted data, constructing output data without prior programming of such information [21]. AI and ML are being increasingly applied in various areas of orthodontics to improve diagnostics, treatment planning, and patient care. Most current applications of this new technology have focused on image analysis and diagnosis [22,23,24,25,26], orthodontic/orthognathic decision-making processes and treatment planning [27,28,29,30,31,32,33,34,35,36,37,38], and growth prediction [39,40]. In an early study to test the ability of AI and ML to predict mandibular growth, Jiwa et al. [41] sought to train a deep learning algorithm to predict mandibular growth. However, none of the landmarks were predicted with an error below 1.5 mm, and only three were predicted with an error below 2.5 mm. In a previously published study from our group, Wood et al. [39] sought to improve the study of Jiwa by gathering a larger number of subjects and reducing the complexity of the algorithm by narrowing the demographics of the subjects, focusing on Class I males. They found that all ML methods tested could accurately predict post-pubertal mandibular length and *Y*-axis within the range of 3.5 mm and 1.5°, respectively. The initial findings of these studies showed promising results in accurately predicting mandibular growth using ML techniques. Gender plays a significant role in human craniofacial growth, with variations observed in the timing and magnitude of growth between males and females [11,42]. Despite this, there is a lack of research evaluating the accuracy of ML models in predicting female pubertal mandibular growth. Hence, the objective of this study was to develop a novel ML model capable of accurately predicting pubertal mandibular growth in Class I females.

## 2. Materials and Methods

### 2.1. Study Sample

The digital lateral cephalometric radiographs used to formulate data for this retrospective study were collected from the American Association of Orthodontists Foundation (AAOF) Craniofacial Growth Legacy Collection [43]. The collection consists of patient radiographic images from the following growth studies: Bolton-Brush Growth, Burlington Growth, Denver Growth, Fels Longitudinal, Forsyth Twin, Iowa Growth, Mathews Growth, Michigan Growth, and Oregon Growth. Inclusion criteria included female subjects with cephalometric radiographs captured during the circumpubertal developmental period and Angle Class I occlusion. Three timepoints were gathered with T1 representing the pre-pubertal stage (mean age SD: 10.05 ± 0.33 yrs), T2 representing the pubertal stage (mean age SD: 11.98 ± 0.36 yrs), and T3 representing the post-pubertal stage (mean age SD: 13.85 ± 0.55 yrs). Subjects exhibiting craniofacial anomalies, noticeable skeletal asymmetries, inadequate image quality, or missing relevant timepoints were excluded from the study. A total of 176 subjects that satisfied the inclusion criteria were selected for the study.

### 2.2. Sample Size Justification

Power analysis revealed that a minimum of 36 subjects in the test set was required to obtain a 95% confidence interval for the intraclass correlation coefficients (ICCs), ranging from 0.64 to 0.89, assuming the ICC is 0.80. Furthermore, higher ICC values would result in narrower confidence interval widths.

### 2.3. Data Collection

**Figure 1:**
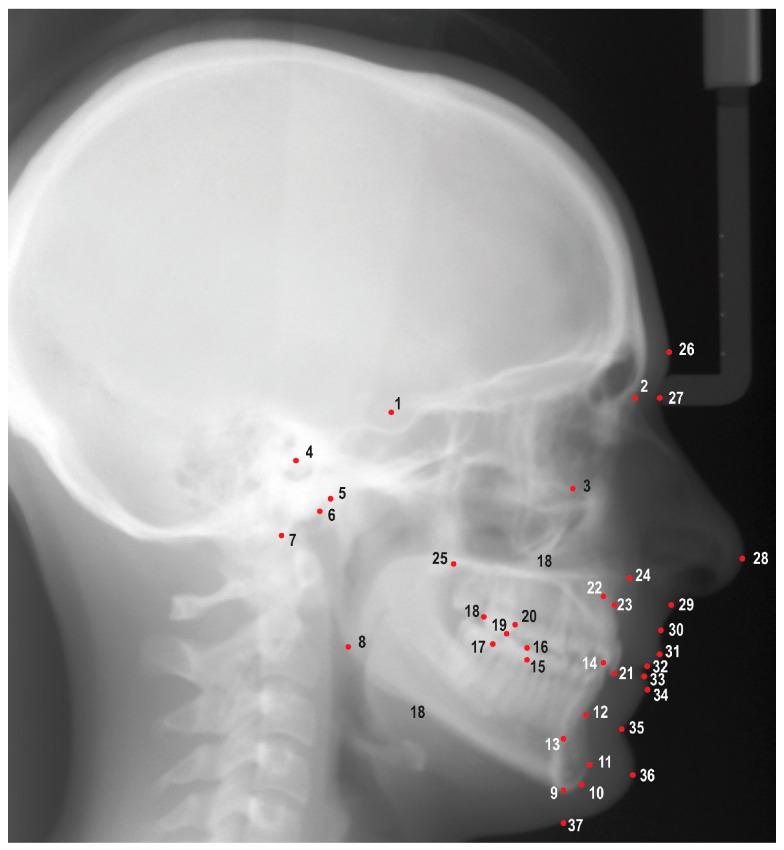
Cephalometric landmarks used in this study. 1. Sella (S), 2. Nasion (N), 3. Orbitale (Or), 4. Porion (Po), 5. Condylion (Co), 6. Articulare (Ar), 7. Basion (Ba), 8. Gonion (Go), 9. Menton (Me), 10. Gnathion (Gn), 11. Pogonion (Pog), 12. B point (B), 13. Lower incisor root apex (L1a), 14. Lower incisor incisal edge (L1i), 15. Mesial of lower first molar (L6m), 16. Mesiobuccal cusp of lower first molar (L6mb), 17. Distal of lower first molar (L6d), 18. Distal of upper first molar (U6d), 19. Mesiobuccal cusp of upper first molar (U6mb), 20. Mesial of upper first molar (U6m), 21. Upper incisor incisal edge (U1i), 22. Upper incisor root apex (U1a), 23. A point (A), 24. Anterior nasal spine (ANS), 25. Posterior nasal spine (PNS), 26. Glabella (G), 27. Soft tissue nasion (N′), 28. Pronasale (Pn), 29. Subnasale (Sn), 30. Soft tissue A point (A’), 31. Upper lip (Ls), 32. Stomion superioris (Ss), 33. Stomion inferioris (Si), 34. Lower lip (Li), 35. Soft tissue B point (B’), 36. Soft tissue pogonion (Pog′), 37. Soft tissue menton (Me′).

Digital images from the AAOF repository were uploaded into Dolphin Imaging v. 11.95 (Dolphin Imaging and Management Solutions, Chatsworth, CA, USA) and were traced by a single investigator (M.P.) using 25 hard tissue landmarks and 12 soft tissue landmarks (Figure 1). A total of 47 linear and angular measurements were measured and recorded; definitions of the measurements are listed in Table S1. Images were scaled by using fiducial data embedded on the images as described in reference material provided by the AAOF. Fiduciaries are reference marks located on the images with known coordinate values allowing the user to compute the scale of the image. The demographic and cephalometric data were subsequently entered into a spreadsheet and securely stored in a cloud service (OneDrive, Microsoft Co., Redmond, WA, USA). To evaluate the repeatability of measurements, a research randomizer was employed to randomly choose 10 images for retracing. The ICCs were utilized to assess the repeatability of these measurements.

### 2.4. Algorithm Training and Testing

**Figure 2:**
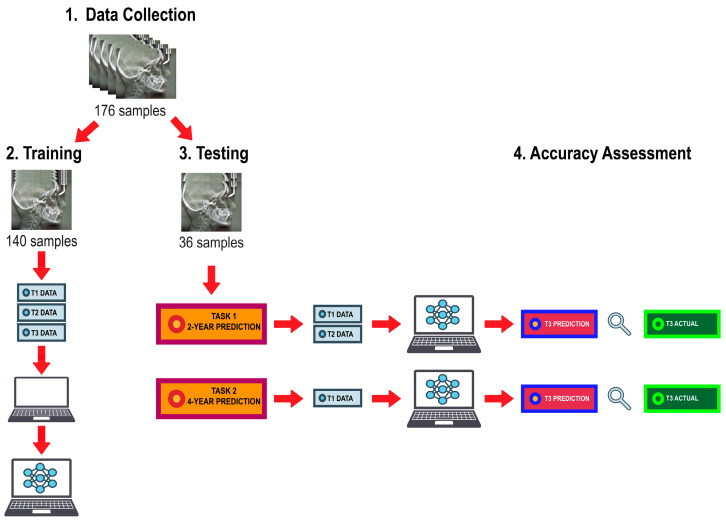
Algorithm training and testing workflow.

The algorithm training and testing workflow is illustrated in Figure 2. Data were randomly distributed into a training set consisting of 80% of the subjects (*n* = 140) and a test set consisting of the remaining 20% (*n* = 36). The training set was used to train the ML models using the linear and angular measurements derived from lateral cephalogram tracings from all three timepoints. The prediction task was executed by giving the algorithms input data from the test set to predict growth magnitude and direction of the mandible at T3. They were first given measurements from T1 and T2 to predict values at T3, then given measurements from T1 alone to predict values at T3, thus, providing a 2-year prediction and a 4-year prediction, respectively. The performance of the trained models was assessed based on their ability to predict post-pubertal mandibular length (Co-Gn) and *Y*-axis (SGn-SN).

Overall, six traditional regression algorithms and a small neural network (NN) model were trained and tested for analysis: XGBoost regression, Random Forest regressor, Lasso, Ridge, Linear Regression, support vector regression (SVR), and multilayer perceptron (MLP) regressor. To explore the linear relationship, least squares regression without any regularizer (Linear Regression) and with L1 (Lasso) and L2 (Ridge) regularizers was implemented. Least squares method is a standard statistical method used to approximate the solution of problems that have more equations than unknowns. For data that did not fall in a linear path, nonlinear methods like kernel-based SVR, tree-based XGBoost, RF, and NN such as MLP are natural choices. Due to small training set and mixture of both numerical and categorical features, tree-based regression methods were first to try, and we explored boosting (XGBoost) and bagging trees (Random Forrest) regressors. Random Forest creates an ensemble of decision trees to minimize the differences between predicted and actual values of dependent variables; thus, it is less likely to overfit training data [44]. Although the training set was too small for data hungry NN model, for the sake of completeness, we added MLP regressor into the training algorithms. The input to all ML models were the values of the 47 covariates, and models were asked to predict mandibular length and *Y*-axis growth at 2 and 4 years. We performed automated hyperparameter tuning using python Hyperopt package for all the models with 100 iterations for each model. The best configuration was chosen based on the score on validation set, and then frozen models were tested.

### 2.5. Statistical Analysis

The mean absolute error (MAE), root mean square error (RMSE), mean error (ME), ICCs, and Bland–Altman plots were calculated for each technique to evaluate the agreement between the predicted and actual outcome measurements. The accuracy percentage of the methods was calculated using the formula (1 − (MAE/Actual value) × 100). The directional and absolute differences between the predicted and actual measurements were calculated and compared between prediction methods using analysis of variance (ANOVA), with random effects to account for data correlation within the 2-year prediction data, within the 4-year prediction data, and overall, and allowed for different error variances for the 2-year and 4-year prediction data. Comparisons of interest were among 2-year predictions for each method, among 4-year predictions for each method, and between 2-year and 4-year predictions by method. Paired *t*-tests were used to test for a significant mean directional difference between predicted and actual measurements. A two-sided 5% significance level was used for all tests. All analyses were performed using SAS version 9.4 (SAS Institute, Inc., Cary, NC, USA).

## 3. Results

### 3.1. Reliability Analysis

The results of the reliability analysis are given in Table S2. Most variables showed excellent repeatability (ICCs > 0.90) [45], with the remainder having good repeatability (0.75 < ICC < 0.90). The two exceptions were soft tissue UFH (G’-Sn) and the Holdaway ratio (L1-NB:Pg-NB) which had poor repeatability (ICCs < 0.50).

### 3.2. Descriptive Statistics

The descriptive statistics of the cephalometric measurements at T1, T2, and T3, including mean, standard deviation, and minimum/maximum values, are shown in Table S3. 

### 3.3. Prediction of the Female Post-Pubertal Mandibular Length

**Figure 3:**
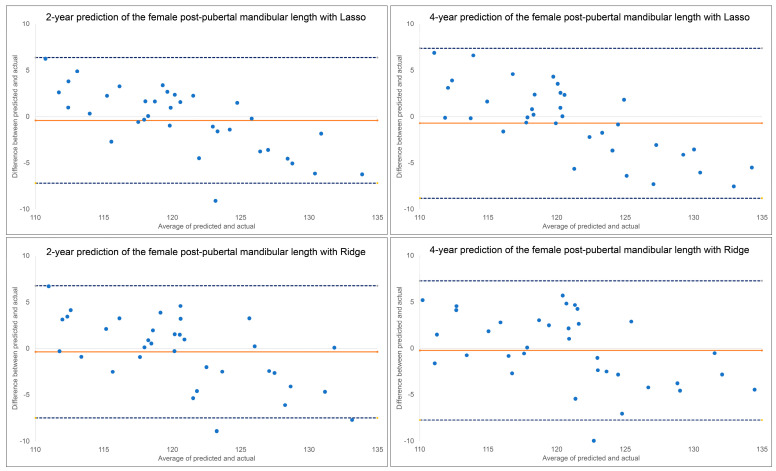
Bland–Altman plots for 2-year and 4-year predictions of female post-pubertal mandibular length using Lasso (**top**) and Ridge (**bottom**). The blue dashed lines represent upper and lower bounds of the 95% confidence intervals. Orange solid line represents mean difference between predicted and actual post-pubertal mandibular length.

The results for the 2-year and 4-year predictions of female post-pubertal mandibular length are given in Table 1 and Figure 3. For the 2-year prediction, MAEs ranged from 2.78 mm to 5.40 mm, with Lasso being most accurate and MLP regressor the least. All methods demonstrated moderate to good correlation between predicted and actual values (0.63 < ICCs < 0.86). Accuracy percentages ranged from 95.56% to 97.63%. For the 4-year prediction, MAEs ranged from 3.21 mm to 4.00 mm, with Ridge being most accurate and Random Forest the least. All methods demonstrated moderate to good correlation between predicted and actual values (0.61 < ICCs < 0.84). Accuracy percentages ranged from 96.71% to 97.36%. 

Bland–Altman plots indicated a discernable pattern between predicted and actual values (Figure 3). Both Lasso and Ridge over-estimated post-pubertal mandibular length for smaller lengths and under-estimated it for larger lengths, in both the 2-year and 4-year predictions. 

**Figure 4:**
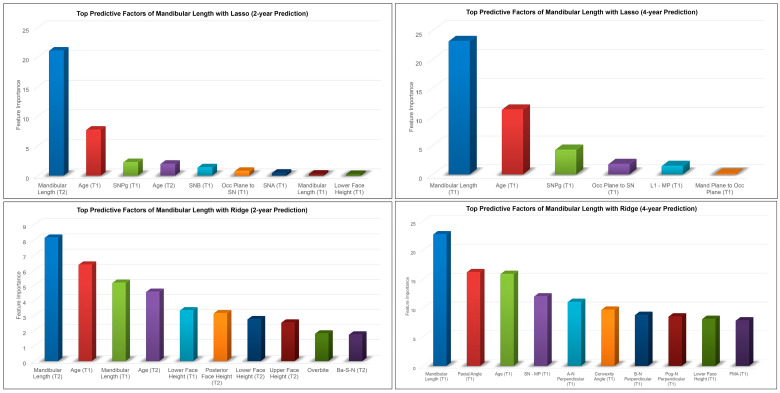
Top predictive factors for 2-year and 4-year predictions of female post-pubertal mandibular length using Lasso (**top**) and Ridge (**bottom**).

The most predictive factors of female post-pubertal mandibular length selected by Lasso and Ridge are presented in Figure 4. Mandibular length, age, SNPg, occlusal plane to mandibular plane, SNB, and L1-MP were among the most predictive factors selected by Lasso, while Ridge additionally used lower, upper, and posterior face heights in its predictions. 

### 3.4. Prediction of the Female Post-Pubertal Y-Axis

**Figure 5:**
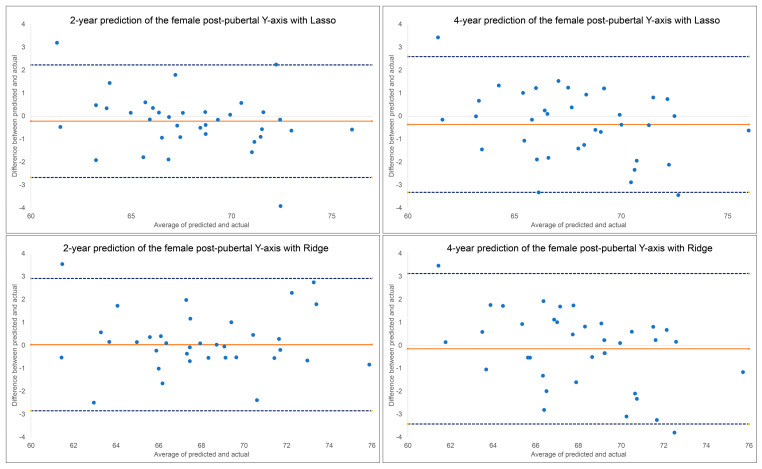
Bland–Altman plots for 2-year and 4-year predictions of female post-pubertal *Y*-axis using Lasso (**top**) and Ridge (**bottom**). The blue dashed lines represent upper and lower bounds of the 95% confidence intervals. Orange solid line represents mean difference between predicted and actual *Y*-axis.

**Figure 6:**
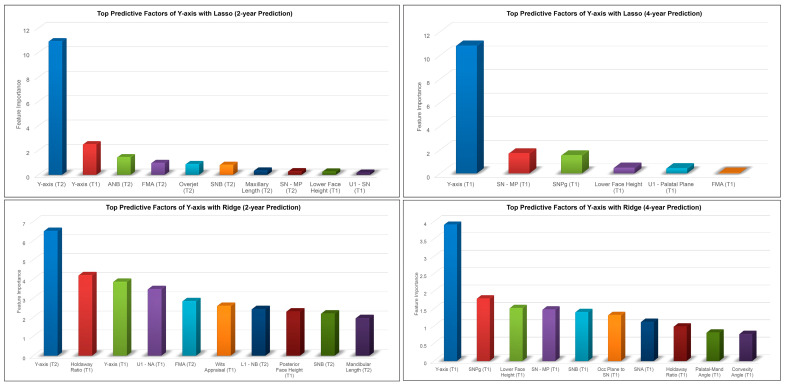
Top predictive factors for 2-year and 4-year predictions of female post-pubertal *Y*-axis using Lasso (**top**) and Ridge (**bottom**).

The results for the 2-year and 4-year predictions of female post-pubertal *Y*-axis are given in Table 2 and Figure 5. For the 2-year prediction, MAEs ranged from 0.88° to 1.48°, with Lasso being most accurate and MLP regressor the least. All methods demonstrated good to excellent correlation between predicted and actual values (0.79 < ICCs < 0.94). Accuracy percentages ranged from 97.83% to 98.71%. For the 4-year prediction, MAEs ranged from 1.19° to 1.66°, with Lasso being most accurate and Random Forest the least. All methods demonstrated good to excellent correlation between predicted and actual values (0.87 < ICCs < 0.90). Accuracy percentages ranged from 97.56% to 98.25%. No discernable pattern was detected for the Bland–Altman plots between predicted and actual values (Figure 5). The most predictive factors of female post-pubertal *Y*-axis selected by Lasso and Ridge are presented in Figure 6. *Y*-axis, ANB, SN-MP, FMA, SN-Pg, and lower face height were among the most predictive factors selected by Lasso, while Ridge additionally used Holdaway ratio, U1-NA, Wits appraisal, and SNB in its predictions.

### 3.5. Method Comparison

Directional and absolute difference comparisons between ML methods for 2-year prediction of post-pubertal mandibular length are given in Table 3. Significant directional and absolute differences were observed among the ML methods in 2-year prediction of the post-pubertal mandibular length (*p* < 0.05). MLP regressor produced significantly different directional results compared to all the tested ML methods; Linear Regression also showed significant directional differences from multiple other methods. MLP regressor and Linear Regression produced estimates that were significantly larger compared to various other ML methods.

Similar results were observed among the ML methods in 4-year prediction of the post-pubertal mandibular length (Table 4). MLP regressor, Random Forest, and XGBoost regression showed significant directional differences compared to various other methods (*p* < 0.05). In terms of absolute differences, MLP regressor and Random Forest produced estimates that were significantly larger than Lasso and Ridge. 

The ML methods had greater agreement for *Y*-axis than mandibular length in both absolute and directional difference and in both the 2-year and 4-year predictions (Table 5 and Table 6). MLP regressor produced estimates significantly larger than Lasso, Ridge, and SVR in the 2-year prediction, while SVR had significant directional difference compared to most other methods in the 4-year prediction (*p* < 0.05).

Lastly, the directional and absolute differences between the 2-year and 4-year predictions of post-pubertal mandibular length and *Y*-axis with each ML algorithm were compared, and the results are given in Table 7 and Table 8. Directional and absolute differences of the mandibular length were significantly smaller in the 4-year predictions compared to the 2-year predictions for Linear Regression (*p* = 0.028 for directional differences, *p* < 0.001 for absolute differences). Absolute differences of *Y*-axis were significantly larger in the 4-year predictions compared to the 2-year predictions for Random Forest (*p* = 0.025) and SVR (*p* = 0.039). No significant differences in directional differences of *Y*-axis were found between the 2-year or 4-year predictions for any method (*p* > 0.05).

## 4. Discussion

A considerable amount of variation in the amount and direction of pubertal mandibular growth exists across genders, races, and individuals [10,13,15,42,46,47]. This fact makes prediction of mandibular growth a very complex process. To achieve accurate and reliable predictions, a stepwise approach is essential, starting with a basic input that lays the foundation for more comprehensive analyses. With that in mind, specific inclusion criteria were employed in this study. Only records from girls at the circumpubertal stage (10 to 14 years) were analyzed to investigate the peak growth and maturation for the average female. Our sample was further narrowed by selecting individuals without significant skeletal sagittal discrepancies, as mandibular growth patterns differ significantly in the presence of sagittal discrepancy. By limiting the initial dataset to this homogeneous group, we can gain valuable insights into the growth patterns specific to this subset. This basic input aids in establishing the fundamental relationships between various variables and mandibular growth within the Class I population. Once the foundation is established, incorporating subjects with Class II and Class III malocclusions into the study will be the next logical step. This expansion introduces greater complexity to the analysis, as these malocclusions present different growth patterns compared to Class I. Furthermore, establishing a baseline reference with restrictive inclusion criteria allows us to gain insight into the fundamental principles, patterns, and trends of utilizing AI predictive technology. This paves the way for more in-depth analysis, hypothesis testing, and the development of advanced methodologies.

There are two major factors that determine the final position of the mandible: mandibular length represents magnitude, and *Y*-axis represents direction of growth. The primary aim of this study was to utilize ML models to accurately predict post-pubertal mandibular length and *Y*-axis from cephalometric data of a subject given data from before (T1) and during (T2) peak height velocity. Predictions using pre-pubertal data alone provide a 4-year forecast of growth, while adding pubertal data provides a 2-year prediction. It would be expected that more input data would result in a more accurate prediction, but it would also be less clinically useful. The majority of the ML models were able to produce 4-year predictions of post-pubertal mandibular length within 4 mm and ICCs > 0.75. The 2-year prediction was marginally improved with two of the ML algorithms predicting mandibular length under 3 mm and ICCs 0.85 or better. For *Y*-axis, all but one of the ML algorithms had 4-year predictions under 1.5° and ICCs 0.84 or better. The 2-year predictions were improved with one ML algorithm predicting *Y*-axis within 0.88° and an ICC of 0.94. Overall, with a few exceptions, the ML algorithms did not produce significantly more accurate predictions of post-pubertal mandibular length and *Y*-axis with the addition of pubertal data. This is a promising finding because an accurate prediction from a single radiograph would be very clinically useful. It would mean fewer radiographic exposures for the patient and less time wasted waiting for more growth to occur. Forecasting growth would allow the orthodontist to decide whether or not growth modification would be required as a part of the treatment plan. 

There are many potential variables that can influence mandibular growth. Previous studies investigating methods to predict mandibular growth have noted this challenge. Skieller et al. [16] identified four variables that could predict mandibular growth rotation and direction. However, Leslie et al. [17] tested their method and found that the values for the four variables could be swapped with random values and produce similar predictions. The ML algorithms in our study identified the features that had the most influence in the process of predicting post-pubertal mandibular length and *Y*-axis. The most influential feature identified by each ML algorithm for predicting each variable was found to be the value of the same variable at the most recent timepoint. It stands to reason that this would be the case and provides proof of concept that the algorithms appropriately weighted predictive factors. The majority of the predictive features for mandibular length were values representing maxillary and mandibular sagittal skeletal bases. The mandibular rotation model was also found to be an important factor. Vertical features carried heavier weight in the 2-year prediction than the 4-year. The fact that vertical growth continues after the completion of sagittal growth might explain this finding. Interestingly, Wood et al. [39] found that vertical features weighed more heavily in their study on Class I males. *Y*-axis predictive features identified by the ML algorithms in both studies were mostly angular measurements related to the mandibular plane and vertical features. This makes sense considering direction of mandibular growth directly relates to lower face height. Predictive features relating to dental values were somewhat more surprising; upper and lower incisor angulation and overjet were also identified by the ML. This could be explained by the fact that the dentition must compensate for skeletal growth patterns. 

A comparison of the ML algorithms revealed very little difference when predicting post-pubertal *Y*-axis. *Y*-axis is less variable over time than mandibular length, making its measurements more predictable. There was also no clear superiority among the ML algorithms in predicting mandibular length. The Ridge and Lasso models most consistently had the best MAEs and ICCs which is why they were chosen to be represented in our predictive feature graphs. Based on the Bland–Altman plots, some of the plots indicate that differences between predicted and actual values have a discernable pattern. Mandibular length was constantly over-estimated for smaller lengths and under-estimated for larger lengths with all ML methods except MLP regression. MLP regression, a neural-network-based model, consistently under-estimated the lengths. No obvious estimation pattern was seen in the *y*-axis predictions. 

The present research study acknowledges several limitations that need to be considered. First, the study relied on retrospective data, which inherently carries the risk of recall bias and limited availability of certain information. Second, given the limited information on subjects provided in the AAOF Legacy Collection, developmental stage was based on chronological age in lieu of other developmental indicators. While this is the least correlative indicator of maturation, it simplified the identification of subjects to be included in the study. Third, the sample size used in this study was relatively small, which may limit the generalizability of the findings to larger populations. Fourth, the study faced challenges in obtaining standardized sources of data, leading to variations in data quality and reliability. Finally, it is important to acknowledge the potential for human error in cephalometric tracing and analysis, which can introduce unintentional biases or inaccuracies. Despite these limitations, the study’s findings provide valuable insights and serve as a starting point for further investigation in this area.

## 5. Conclusions

The tested ML models were able to predict post-pubertal mandibular length within 3 mm and *Y*-axis within 1° and did not produce significantly more accurate predictions with the addition of pubertal data. Most predictive factors for mandibular length were mandibular length at previous timepoints, age, sagittal positions of the maxillary and mandibular skeletal bases, mandibular plane angle, and anterior and posterior face heights. Most predictive factors for *Y*-axis were *Y*-axis at previous timepoints, mandibular plane angle, and sagittal positions of the maxillary and mandibular skeletal bases. All ML algorithms yielded consistent results with the exception of MLP regressor consistently underestimating the mandibular length.

## Figures and Tables

**Table 1:** Results of 2-year and 4-year prediction of the female post-pubertal mandibular length.

	2-Year Prediction					4-Year Prediction				
Models	MAE	RMSE	ME	ICC	Accuracy %	MAE	RMSE	ME	ICC	Accuracy %
XGBoost	3.10	4.18	0.75	0.79	97.45	3.97	5.04	1.17	0.70	96.73
Random Forest	3.16	4.19	0.70	0.74	97.40	4.00	5.31	1.55	0.61	96.71
Lasso	2.78	3.46	0.47	0.86	97.71	3.25	4.13	0.71	0.79	97.33
Ridge	2.88	3.60	0.35	0.85	97.63	3.21	3.79	0.23	0.84	97.36
Linear Regression	5.40	6.40	−1.13	0.63	95.56	3.53	4.21	0.13	0.81	97.10
SVR	3.25	3.85	0.69	0.84	97.33	3.74	4.40	0.79	0.78	96.92
MLP	3.88	5.24	1.39	0.63	96.81	3.78	4.65	−2.11	0.73	96.89

MAE: Mean absolute error, RMSE: Root mean square error, ME: Mean error, ICC: Intra-class correlation coefficient.

**Table 2:** Results of 2-year and 4-year prediction of the female post-pubertal *Y*-axis.

	2-Year Prediction					4-Year Prediction				
Models	MAE	RMSE	ME	ICC	Accuracy %	MAE	RMSE	ME	ICC	Accuracy %
XGBoost	1.12	1.43	0.34	0.91	98.36	1.37	1.64	0.46	0.89	97.99
Random Forest	1.24	1.54	0.52	0.90	98.18	1.66	2.04	0.65	0.84	97.56
Lasso	0.88	1.25	0.22	0.94	98.71	1.19	1.53	0.36	0.90	98.25
Ridge	1.01	1.45	−0.04	0.92	98.52	1.32	1.65	0.15	0.88	98.06
Linear Regression	1.2	1.52	0.19	0.91	98.24	1.4	1.71	0.44	0.89	97.95
SVR	1.01	1.34	0.04	0.93	98.52	1.43	1.75	−0.12	0.87	97.90
MLP	1.48	2.42	0.52	0.79	97.83	1.43	1.76	−0.27	0.87	97.90

MAE: Mean absolute error, RMSE: Root mean square error, ME: Mean error, ICC: Intra-class correlation coefficient.

**Table 3:** Directional and absolute difference comparisons between ML methods for 2-year prediction of post-pubertal mandibular length.

Directional Difference		Absolute Difference	
Result	*p*-Value	Result	*p*-Value
Lasso < Linear Regression	0.01	Lasso < Linear Regression	<0.001
Lasso > MLP	<0.001	Lasso < MLP	<0.001
Lasso and Random Forest	0.27	Lasso and Random Forest	0.15
Lasso and Ridge	0.93	Lasso and Ridge	0.80
Lasso and SVR	0.63	Lasso and SVR	0.30
Lasso and XGBoost	0.56	Lasso and XGBoost	0.48
Linear Regression > MLP	<0.001	Linear Regression and MLP	0.10
Linear Regression > Random Forest	<0.001	Linear Regression > Random Forest	<0.001
Linear Regression > Ridge	0.014	Linear Regression > Ridge	<0.001
Linear Regression > SVR	0.003	Linear Regression > SVR	<0.001
Linear Regression > XGBoost	0.002	Linear Regression > XGBoost	<0.001
MLP < Random Forest	<0.001	MLP > Random Forest	0.014
MLP < Ridge	<0.001	MLP > Ridge	<0.001
MLP < SVR	<0.001	MLP > SVR	0.004
MLP < XGBoost	<0.001	MLP > XGBoost	0.001
Random Forest and Ridge	0.23	Random Forest and Ridge	0.23
Random Forest and SVR	0.53	Random Forest and SVR	0.68
Random Forest and XGBoost	0.59	Random Forest and XGBoost	0.45
Ridge and SVR	0.57	Ridge and SVR	0.43
Ridge and XGBoost	0.51	Ridge and XGBoost	0.65
SVR and XGBoost	0.92	SVR and XGBoost	0.74

**Table 4:** Directional and absolute difference comparisons between ML methods for 4-year prediction of post-pubertal mandibular length.

Directional Difference		Absolute Difference	
Result	*p*-Value	Result	*p*-Value
Lasso and Linear Regression	0.20	Lasso and Linear Regression	0.51
Lasso > MLP	<0.001	Lasso < MLP	0.04
Lasso > Random Forest	0.04	Lasso and Random Forest	0.05
Lasso and Ridge	0.29	Lasso and Ridge	0.93
Lasso and SVR	0.86	Lasso and SVR	0.25
Lasso and XGBoost	0.30	Lasso and XGBoost	0.09
Linear Regression > MLP	<0.001	Linear Regression and MLP	0.16
Linear Regression > Random Forest	0.001	Linear Regression and Random Forest	0.19
Linear Regression and Ridge	0.82	Linear Regression and Ridge	0.45
Linear Regression and SVR	0.15	Linear Regression and SVR	0.61
Linear Regression > XGBoost	0.02	Linear Regression and XGBoost	0.29
MLP < Random Forest	0.004	MLP and Random Forest	0.91
MLP < Ridge	<0.001	MLP > Ridge	0.03
MLP < SVR	<0.001	MLP and SVR	0.37
MLP < XGBoost	<0.001	MLP and XGBoost	0.72
Random Forest < Ridge	0.002	Random Forest > Ridge	0.04
Random Forest and SVR	0.06	Random Forest and SVR	0.43
Random Forest and XGBoost	0.30	Random Forest and XGBoost	0.80
Ridge and SVR	0.22	Ridge and SVR	0.21
Ridge > XGBoost	0.04	Ridge and XGBoost	0.07
SVR and XGBoost	0.39	SVR and XGBoost	0.58

**Table 5:** Directional and absolute difference comparisons between ML methods for 2-year prediction of post-pubertal *Y*-axis.

Directional Difference		Absolute Difference	
Result	*p*-Value	Result	*p*-Value
Lasso and Linear Regression	0.92	Lasso and Linear Regression	0.12
Lasso and MLP	0.26	Lasso < MLP	0.004
Lasso and Random Forest	0.42	Lasso and Random Forest	0.13
Lasso and Ridge	0.33	Lasso and Ridge	0.53
Lasso and SVR	0.50	Lasso and SVR	0.53
Lasso and XGBoost	0.65	Lasso and XGBoost	0.26
Linear Regression and MLP	0.22	Linear Regression and MLP	0.19
Linear Regression and Random Forest	0.37	Linear Regression and Random Forest	0.97
Linear Regression and Ridge	0.38	Linear Regression and Ridge	0.36
Linear Regression and SVR	0.56	Linear Regression and SVR	0.35
Linear Regression and XGBoost	0.58	Linear Regression and XGBoost	0.67
MLP and Random Forest	0.74	MLP and Random Forest	0.17
MLP < Ridge	0.04	MLP > Ridge	0.03
MLP and SVR	0.07	MLP > SVR	0.03
MLP and XGBoost	0.50	MLP and XGBoost	0.08
Random Forest and Ridge	0.08	Random Forest and Ridge	0.37
Random Forest and SVR	0.14	Random Forest and SVR	0.37
Random Forest and XGBoost	0.73	Random Forest and XGBoost	0.70
Ridge and SVR	0.76	Ridge and SVR	0.99
Ridge and XGBoost	0.15	Ridge and XGBoost	0.61
SVR and XGBoost	0.26	SVR and XGBoost	0.61

**Table 6:** Directional and absolute difference comparisons between ML methods for 4-year prediction of post-pubertal *Y*-axis.

Directional Difference		Absolute Difference	
Result	*p*-Value	Result	*p*-Value
Lasso and Linear Regression	0.69	Lasso and Linear Regression	0.20
Lasso and MLP	0.65	Lasso and MLP	0.15
Lasso and Random Forest	0.15	Lasso < Random Forest	0.005
Lasso and Ridge	0.29	Lasso and Ridge	0.43
Lasso < SVR	0.02	Lasso and SVR	0.14
Lasso and XGBoost	0.63	Lasso and XGBoost	0.27
Linear Regression and MLP	0.40	Linear Regression and MLP	0.89
Linear Regression and Random Forest	0.29	Linear Regression and Random Forest	0.13
Linear Regression and Ridge	0.15	Linear Regression and Ridge	0.61
Linear Regression < SVR	0.006	Linear Regression and SVR	0.86
Linear Regression and XGBoost	0.93	Linear Regression and XGBoost	0.85
MLP and Random Forest	0.06	MLP and Random Forest	0.17
MLP and Ridge	0.54	MLP and Ridge	0.52
MLP and SVR	0.05	MLP and SVR	0.98
MLP and XGBoost	0.35	MLP and XGBoost	0.74
Random Forest < Ridge	0.012	Random Forest > Ridge	0.045
Random Forest < SVR	<0.001	Random Forest and SVR	0.18
Random Forest and XGBoost	0.33	Random Forest and XGBoost	0.09
Ridge and SVR	0.18	Ridge and SVR	0.50
Ridge and XGBoost	0.13	Ridge and XGBoost	0.75
SVR > XGBoost	0.004	SVR and XGBoost	0.72

**Table 7:** Comparisons of the directional and absolute differences between the 2-year and 4-year predictions of post-pubertal mandibular length.

	Directional Difference		Absolute Difference	
Method	Result	*p*-Value	Result	*p*-Value
XGBoost	2-year and 4-year	0.45	2-year and 4-year	0.06
Random Forest	2-year and 4-year	0.31	2-year and 4-year	0.18
Lasso	2-year and 4-year	0.59	2-year and 4-year	0.29
Ridge	2-year and 4-year	0.83	2-year and 4-year	0.48
Linear Regression	2-year > 4-year	0.03	2-year > 4-year	<0.001
SVR	2-year and 4-year	0.86	2-year and 4-year	0.30
MLP	2-year and 4-year	0.30	2-year and 4-year	0.28

**Table 8:** Comparisons of the directional and absolute differences between the 2-year and 4-year predictions of post-pubertal *Y*-axis.

	Directional Difference		Absolute Difference	
Method	Result	*p*-Value	Result	*p*-Value
XGBoost	2-year and 4-year	0.66	2-year and 4-year	0.21
Random Forest	2-year and 4-year	0.41	2-year < 4-year	0.025
Lasso	2-year and 4-year	0.60	2-year and 4-year	0.13
Ridge	2-year and 4-year	0.50	2-year and 4-year	0.13
Linear Regression	2-year and 4-year	0.36	2-year and 4-year	0.32
SVR	2-year and 4-year	0.54	2-year < 4-year	0.039
MLP	2-year and 4-year	0.36	2-year and 4-year	0.81

## Data Availability

The data underlying this article are available in the article. The datasets were derived from sources in the public domain from the AAOF Legacy Collection at https://www.aaoflegacycollection.org (accessed on 25 April 2023).

## Supplementary Materials

The following supporting information can be downloaded at https://www.mdpi.com/article/10.3390/diagnostics13172729/s1, Table S1: Cephalometric variables and their definitions. Table S2: Intra-examiner repeatability of the measurements. Table S3: The descriptive statistics of the cephalometric measurements at T1, T2, and T3, including mean, standard deviation, and minimum/maximum values.
