# Supplementary material for: Short- and Long-Term Prediction of the Post-Pubertal Mandibular Length and Y-Axis in Females Utilizing Machine Learning
Source: Diagnostics (Basel). 2023 Aug 22;13(17):2729. doi: 10.3390/diagnostics13172729 (PMC10486405; doi:10.3390/diagnostics13172729)
Supplement: Supplementary file 1 [file diagnostics-13-02729-s001.zip › diagnostics-2506005-supplementary.pdf]

Supplementary Table S1. Cephalometric variables and their definitions.

Category	Measurements	Definitions
Maxilla to Cranial Base	SNA (°)	The angle formed by connecting sella, nasion, and A-point
	SN-Palatal Plane (°)	The angle formed from the intersection of sella-nasion line and a line drawn connecting anterior nasal spine to posterior nasal spine
	SN-Occlusal Plane (°)	The angle formed from sella-nasion and occlusal plane
	A-N Perpendicular (mm)	The linear distance from A point to the nasion perpendicular.
Mandible to Cranial Base	SNB (°)	The angle formed by connecting sella, nasion, and B-point
	SNPg (°)	The angle formed by connecting sella, naison, and pogonion
	FMA: MP-FH (°)	The angle formed from the intersection of porion-orbitale line and a line drawn connecting gonion to gnathion
	SN-MP (°)	The angle formed from the intersection of sella-nasion line and a line drawn connecting gonion to gnathion
	Mandibular Plane to Occlusal Plane (°)	The angle formed by mandibular plane and occlusal plane
	B-N Perpendicular (mm)	The linear distance from B point to nasion perpendicular
	Pog-N Perpendicular (mm)	The linear distance from pogonion to nasion perpendicular
	Y-Axis: SGn-SN (°)	The angle formed by connecting nasion, sella, and gnathion
Maxilla to Mandible	ANB (°)	The difference between SNA and SNB
	Palatal-Mandibular Angle (PP-MP) (°)	The angle formed from palatal plane and mandibular plane
	Wits Appraisal (mm)	The distance between A point to Occlusal plane and B point to Occlusal plane
	Maxillary Length: ANS-PNS (mm)	The linear measurement between anterior nasal spine and posterior nasal spine
	Mandibular Length: Co-Gn (mm)	The linear measurement between condylion and gnathion
Cranial Base	Cranial Base Flexure Angle: Ba-S-N (°)	The angle formed by connecting basion, sella, and nasion
Upper Incisors to Maxilla	U1-SN (°)	The angle formed from a line connecting sella to nasion and a line connecting the upper incisor incisal tip to the root apex
	U1-NA (°)	The angle formed from a line connecting nasion to A-point and a line connecting the upper incisor incisal tip to the root apex
	U1-NA (mm)	The linear measurement from the labial surface of the upper incisor to the line connecting nasion to A-point
	U1-Palatal Plane (°)	The angle formed by the position of maxillary incisor to palatal plane
	U1 Protrusion (U1-APo) (mm)	The distance from maxillary incisor to the A point-pogonion reference line
Lower Incisors to Mandible	L1-MP (°)	The angle formed from a line connecting the lower incisor incisal tip to the root apex and a line connecting gonion to gnathion
	L1-NB (°)	The angle formed from a line connecting the lower incisor incisal tip to the root apex and a line connecting nasion to B-point
	L1-NB (mm)	The linear measurement from the labial surface of the lower incisor incisal to the line connecting nasion to B-point
	L1 Protrusion (L1-Apo) (mm)	The distance from mandibular incisor to the A point-pogonion reference line
Incisors to Each Other	Interincisal Angle (°)	The angle formed from a line connecting the lower incisor incisal tip to the apex and a line connecting the upper incisor incisal tip to the root apex
	Overjet (mm)	The horizontal distance from maxillary incisor tip to mandibular incisor tip
	Overbite (mm)	The vertical distance from maxillary incisor tip to mandibular incisor tip
Soft Tissue	Upper Lip to E-Plane (mm)	The linear distance from upper lip to a line connecting soft tissue pogonion and pronasale
	Lower Lip to E-Plane (mm)	The linear distance from lower lip to a line connecting soft tissue pogonion and pronasale
	ILG (HP) (mm)	The vertical distance from stomion superius to stomion inferius
	Nasolabial Angle (Pn-Sn-UL) (°)	The angle formed by pronasale, subnasale, and upper lip
	H-Angle (Pg’UL-Pg’Na’) (°)	The angle formed by soft tissue pogonion-upper lip to soft tissue pogonion-soft tissue nasion

	Facial Height (Na'-Me') (mm)	The linear measurement from soft tissue nasion and soft tissue menton
	Soft Tissue Upper Face Height: G'-Sn' (mm)	The linear measurement between soft tissue glabella and soft tissue subnasale
	Soft Tissue Lower Face Height: Sn'-Me' (mm)	The linear measurement between soft tissue subnasale and soft tissue pogonion
	Hard Tissue Upper Face Height: N-ANS (mm)	The linear measurement between nasion and anterior nasal spine
	Hard Tissue Lower Face Height: ANS-Me (mm)	The linear measurement between anterior nasal spine and menton
	UFH (N-ANS/(N-ANS+ANS-Me)) (%)	The ratio of the upper face height to facial height
	LFH (ANS-Me/(N-ANS+ANS-Me)) (%)	The ratio of lower face heigh to facial height
	Posterior Face Height: Ar-Go (mm)	The linear measurement between articulare and gonion
	PFH:AFH (Co-Go:N-Me) (%)	The ratio of posterior facial height to anterior facial height
Profile	Convexity: NA-APo (˚)	The angle formed by connecting nasion, A-point, and pogonion
	Facial Angle: FH-NPo (˚)	The angle formed from a line connecting porion to orbitale and a line connecting nasion to pogonion
	Holdaway Ratio (L1-NB:Pg-NB) (%)	The ratio of the linear distance from the lower incisor to the NB line to the linear distance of pogonion to the NB line
	Holdaway Angle (NB to H-line) (°)	The angle formed by the H-line to NB line

Supplementary Table S2. Intra-examiner repeatability of the measurements.

Measurements	Mean	SD	SE	p-value	ICC
SNA	-0.28	0.75	0.24	0.269	0.97
SN-Palatal Plane	-0.21	0.66	0.21	0.343	0.97
Occlusal Plane to SN	-0.30	0.96	0.30	0.348	0.98
A-N Perpendicular (mm)	-0.17	0.58	0.18	0.381	0.98
SNB	-0.14	0.85	0.27	0.613	0.97
SNPg	-0.39	1.06	0.34	0.275	0.95
FMA (MP-FH)	0.08	0.96	0.30	0.798	0.99
SN - MP	0.31	1.52	0.48	0.534	0.97
Mandibular Plane to Occlusal Plane	0.66	0.88	0.28	0.041	0.97
B-N Perpendicular (mm)	-0.03	0.95	0.30	0.922	0.99
Pog-N Perpendicular (mm)	-0.59	1.25	0.40	0.170	0.98
Y-Axis (SGn-SN)	0.24	0.66	0.21	0.277	0.98
ANB	-0.15	0.24	0.08	0.081	0.99
Palatal-Mandibular Angle (PP-MP)	0.58	0.94	0.30	0.082	0.98
Wits Appraisal (mm)	0.17	0.49	0.16	0.303	0.98
Maxillary length (ANS-PNS) (mm)	0.19	1.60	0.51	0.717	0.89
Mandibular length (Co-Gn) (mm)	-0.43	2.33	0.74	0.574	0.97
Ba-S-N	-0.54	1.40	0.44	0.255	0.95
U1 - SN	0.25	2.30	0.73	0.739	0.92
U1 - NA	0.54	2.11	0.67	0.439	0.95
U1 - NA (mm)	0.18	0.53	0.17	0.311	0.95
U1 - Palatal Plane	0.04	2.06	0.65	0.952	0.95
U1 Protrusion (U1-APo) (mm)	0.23	0.71	0.23	0.336	0.96
L1 - MP	0.95	1.55	0.49	0.084	0.96
L1 - NB	1.02	1.72	0.54	0.094	0.95
L1 - NB (mm)	-0.05	0.59	0.19	0.794	0.96

L1 Protrusion (L1-APo) (mm)	0.17	0.74	0.23	0.485	0.87
Interincisal Angle (U1-L1)	-1.39	2.99	0.95	0.176	0.92
Overjet	-0.02	0.16	0.05	0.705	0.99
Overbite	-0.67	0.69	0.22	0.013	0.88
Upper Lip to E-Plane (mm)	0.13	1.23	0.39	0.746	0.89
Lower Lip to E-Plane (mm)	0.24	1.06	0.33	0.491	0.92
ILG (HP) (mm)	0.35	0.39	0.12	0.019	0.97
Nasolabial Angle (Col-Sn-UL)	3.09	3.63	1.15	0.025	0.85
H-Angle (Pg'UL-Pg'Na')	0.24	1.89	0.63	0.709	0.93
UFH (G'-Sn') (mm)	1.36	6.89	2.18	0.548	0.35
LFH (Sn'-Me') (mm)	-0.32	1.26	0.40	0.444	0.94
Upper Face Height (N-ANS) (mm)	-0.06	0.64	0.20	0.774	0.99
Lower Face Height (ANS-Me) (mm)	-0.41	1.03	0.33	0.240	0.92
UFH (N-ANS/(N-ANS+ANS-Me)) (%)	0.15	0.36	0.11	0.224	0.99
LFH (ANS-Me/(N-ANS+ANS-Me)) (%)	-0.15	0.36	0.11	0.224	0.99
Posterior Face Height (Ar-Go) (mm)	0.84	2.36	0.75	0.290	0.94
PFH:AFH (Co-Go : N-Me) (%)	0.00	1.46	0.46	1.000	0.95
Convexity (NA-APo)	0.19	0.89	0.28	0.518	0.99
Facial Angle (FH-NPo)	-0.28	0.65	0.20	0.204	0.98
Holdaway Ratio (L1-NB:Pg-NB) (%)	-11.09	35.29	11.16	0.346	0.00
Holdaway Angle (NB to H-line)	0.20	1.79	0.56	0.731	0.94

SD: Standard deviation, SE: standard error, ICC: intraclass correlation coefficient.

Supplementary Table S3. The descriptive statistics of the cephalometric measurements at T1, T2, and T3, including mean, standard deviation, and minimum/maximum values.

	T1				T2				T3			
**Measurements**	**Mean**	**SD**	**Min**	**Max**	**Mean**	**SD**	**Min**	**Max**	**Mean**	**SD**	**Min**	**Max**
SNA	79.58	3.12	70.50	87.20	80.01	3.36	71.70	89.50	80.10	3.27	71.60	88.40
SN-Palatal Plane	8.58	2.89	-2.30	14.20	8.46	3.03	-0.70	16.30	8.44	3.10	-0.30	16.20
Occlusal Plane to SN	19.41	3.65	10.60	29.40	18.13	4.14	7.10	27.20	16.97	4.22	4.70	25.50
A-N Perpendicular (mm)	-2.06	3.44	-12.70	8.90	-1.69	3.26	-12.40	5.70	-1.64	3.66	-15.50	6.70
SNB	76.45	3.09	67.90	85.10	77.34	3.24	69.80	88.00	77.76	3.41	69.60	87.40
SNPg	77.12	3.19	68.80	85.20	78.15	3.36	70.50	87.60	78.85	3.53	70.90	87.40
FMA (MP-FH)	28.34	4.40	18.40	39.90	27.49	4.39	16.90	38.40	26.88	4.81	14.40	41.00
SN - MP	34.22	4.47	23.00	46.50	33.43	4.72	21.30	47.00	32.65	5.10	19.20	46.90
Mandibular Plane to Occlusal Plane	15.81	2.99	8.90	23.00	16.24	3.11	8.60	23.70	16.70	3.68	7.30	26.20
B-N Perpendicular (mm)	-8.38	5.39	-23.70	5.20	-7.18	5.16	-24.90	5.80	-6.71	5.97	-28.10	8.40
Pog-N Perpendicular (mm)	-8.30	6.06	-24.20	5.60	-6.65	5.95	-26.60	8.10	-5.54	6.79	-29.00	10.20
Y-Axis (SGn-SN)	68.19	3.11	58.90	76.20	67.95	3.28	57.40	76.00	67.85	3.48	58.40	76.30
ANB	3.13	1.94	-2.80	7.60	2.68	1.94	-2.60	7.50	2.33	2.12	-3.00	8.00
Palatal-Mandibular Angle (PP-MP)	26.65	4.31	13.10	37.70	25.92	4.50	13.80	40.30	25.23	4.88	12.90	39.70
Wits Appraisal (mm)	-0.74	2.36	-7.30	4.40	-0.93	2.49	-8.80	5.90	-0.81	2.92	-8.70	5.10
Maxillary length (ANS-PNS) (mm)	48.79	3.15	41.70	57.50	50.53	3.35	42.80	59.90	52.22	3.61	39.20	61.20
Mandibular length (Co-Gn) (mm)	110.43	6.09	95.50	135.60	115.68	6.58	101.40	133.60	120.71	6.75	103.50	138.90

Ba-S-N	129.71	4.15	120.90	141.70	129.74	4.19	120.70	141.40	129.89	4.51	118.90	141.10
U1 - SN	103.17	6.23	84.80	120.80	103.32	6.49	88.50	124.30	103.25	6.91	85.30	123.20
U1 - NA	23.59	5.67	7.70	42.70	23.31	6.16	8.50	39.10	23.15	6.53	4.70	38.10
U1 - NA (mm)	4.16	1.95	-1.00	11.60	4.61	2.34	-1.60	11.40	5.01	2.54	-2.50	12.00
U1 - Palatal Plane	111.75	5.80	95.70	128.60	111.77	5.96	97.70	129.60	111.69	6.12	97.00	129.20
U1 Protrusion (U1-APo) (mm)	6.03	1.81	1.90	10.30	6.10	2.10	1.10	11.90	6.05	2.27	-0.10	11.90
L1 - MP	90.54	5.98	75.10	106.80	90.63	6.21	73.30	106.90	90.42	6.45	74.10	111.10
L1 - NB	23.63	5.61	6.20	36.10	23.80	5.96	7.60	37.70	23.41	5.88	8.30	40.20
L1 - NB (mm)	4.36	1.79	-0.60	8.80	4.40	1.96	-0.80	9.70	4.52	2.08	-0.80	9.70
L1 Protrusion (L1-APo) (mm)	2.02	1.85	-2.20	6.90	2.14	1.91	-2.60	8.00	2.19	2.02	-2.50	8.50
Interincisal Angle (U1-L1)	129.65	8.10	107.00	149.60	130.21	8.91	109.80	158.70	131.10	9.15	108.10	154.20
Overjet	4.11	1.53	-3.30	8.10	4.11	1.35	0.80	8.70	4.00	1.30	1.10	9.00
Overbite	1.58	1.99	-6.20	5.50	2.12	1.73	-2.40	5.70	1.97	1.85	-6.20	5.60
Upper Lip to E-Plane (mm)	-2.47	2.18	-8.60	3.40	-3.15	2.19	-9.30	1.70	-4.11	2.33	-10.30	3.80
Lower Lip to E-Plane (mm)	-0.30	2.20	-5.10	4.20	-0.83	2.57	-6.50	5.50	-1.54	2.44	-7.80	4.80
ILG (HP) (mm)	2.87	2.84	0.50	13.90	2.78	2.72	0.40	13.60	2.37	2.35	0.50	11.80
Nasolabial Angle (Col-Sn-UL)	115.28	9.64	83.90	141.20	115.43	8.42	85.80	133.80	113.15	8.68	87.30	130.90
H-Angle (Pg'UL-Pg'Na')	14.96	4.13	4.60	28.20	14.81	4.03	0.90	22.20	13.98	4.14	3.80	25.40
UFH (G'-Sn') (mm)	68.05	6.38	53.40	91.10	70.94	6.37	55.40	93.80	72.64	6.02	57.20	87.60
LFH (Sn'-Me') (mm)	66.22	4.38	54.70	76.40	69.10	5.18	56.10	83.20	71.00	4.86	59.00	83.60
Upper Face Height (N-ANS) (mm)	49.92	3.38	41.70	61.30	52.28	3.49	43.30	63.40	53.92	3.40	43.80	66.80
Lower Face Height (ANS-Me) (mm)	61.18	4.03	52.00	71.40	63.22	4.43	53.10	77.40	65.63	4.90	51.70	78.10
UFH (N-ANS/(N-ANS+ANS-Me)) (%)	44.94	1.89	38.70	49.90	45.27	1.86	39.10	49.20	45.15	1.99	39.70	50.50
LFH (ANS-Me/(N-ANS+ANS-Me)) (%)	55.06	1.89	50.10	61.30	54.73	1.86	50.80	60.90	54.88	1.95	49.90	60.30
Posterior Face Height (Ar-Go) (mm)	40.73	3.69	32.40	54.80	43.34	4.00	33.70	54.20	45.91	4.17	36.40	56.20
PFH:AFH (Co-Go : N-Me) (%)	51.91	3.37	44.10	61.40	52.47	3.57	43.60	61.90	53.41	3.93	43.80	64.90
Convexity (NA-APo)	5.23	4.93	-11.40	16.80	3.95	5.06	-10.30	18.20	2.63	5.41	-11.50	17.00
Facial Angle (FH-NPo)	85.42	3.31	77.20	93.30	86.50	3.12	76.90	94.50	87.20	3.40	75.90	95.30
Holdaway Ratio (L1-NB:Pg-NB) (%)	-1.95	33.54	-212.90	253.80	-0.17	22.84	-286.60	32.00	2.47	31.63	-129.60	314.10
Holdaway Angle (NB to H-line)	11.57	4.50	-0.10	24.40	11.10	4.47	-1.80	19.20	9.78	4.60	-1.00	21.50

SD: Standard deviation, Min: minimum, Max: maximum.
